# Effect of acupuncture at ST36 on motor cortical excitation and inhibition

**DOI:** 10.1002/brb3.1370

**Published:** 2019-07-30

**Authors:** Zhong‐Guang Sun, Yan‐Ling Pi, Jian Zhang, Miao Wang, Jun Zou, Wei Wu

**Affiliations:** ^1^ School of Kinesiology Shanghai University of Sport Shanghai China; ^2^ Shanghai Punan Hospital of Pudong New District Shanghai China; ^3^ Affiliated Competitive Sport School Shanghai University of Sport Shanghai China

**Keywords:** Deqi, long‐interval intracortical inhibition, motor‐evoked potential, short‐interval intracortical inhibition, ST36, transcranial magnetic stimulation

## Abstract

**Background:**

Acupuncture at Zusanli (ST36) is often used to facilitate motor recovery after stroke. However, the effect of acupuncture at ST36 on motor cortical excitation and inhibition remains unclear. This study aimed to explore the effect of acupuncture at ST36 on motor cortical excitation and inhibition.

**Methods:**

Twenty healthy volunteers were recruited to receive acupuncture treatment. We selected the acupoint ST36 and its respective sham point as the experimental acupoint. Transcranial magnetic stimulation (TMS) was used to measure motor‐evoked potentials (MEP) at 7 time points—before acupuncture (Pre), acupuncture (T0), 4 and 8 min after acupuncture (T4; T8), needle removal (T12), 4 and 8 min after needle removal (T16; T20). Simultaneously, paired TMS (pTMS) was employed to measure short‐ and long‐interval intracortical inhibition (SICI [short latency intracortical inhibition]; LICI [long latency intracortical inhibition]), respectively, at three time points—before acupuncture (Pre), acupuncture (T0), needle removal (T12). After removing the acupuncture needle, all subjects were asked to quantify their Deqi sensation using a Gas table.

**Results:**

The average Deqi sensation score of all subjects during acupuncture at ST36 was higher than that observed at the sham point. With acupuncture at ST36, the MEP amplitude was higher at three time points (T0, T4, T8) than at Pre, although the MEP amplitude tended toward Pre after needle removal. The MEP amplitude was also higher at the same time points (T0, T4, T8) than at the sham point. Furthermore, the Deqi sensation score was correlated with MEP amplitude. With acupuncture at ST36, SICI and LICI at T0 were higher than those at Pre, and SICI and LICI at T0 were higher than those at the sham point.

**Conclusion:**

Acupuncture at ST36 increased motor cortical excitation and had an effect on the remaining needle phase. Deqi sensation was correlated with MEP amplitude. Acupuncture at ST36 also decreased motor cortical inhibition.

## 1 INTRODUCTION

Acupuncture is an important therapeutic technique in traditional Chinese medicine (TCM). In clinical practice, acupuncture is used to treat various disorders, such as chronic pain syndrome, nausea, vomiting, drug addiction, stroke and asthma (Kaptchuk, 2002). The selection of acupuncture points is based on experiences treating disease in TCM, as more than 300 acupoints have been described. Zusanli (ST36) is an important acupoint; especially used to treat stroke, pain, hypertension and other physiological dysfunctions (Geng et al., 2013). Acupuncture stimulation elicits Deqi sensation, which is a composite of distinct sensations, including soreness, numbness, fullness, heaviness, and dull pain. Deqi sensation may be a useful clinical indicator of effective treatment in TCM.

Neuroimaging has provided revolutionary tools to monitor the response of the brain to acupuncture with specific regional localization. Acupuncture at ST36 was shown to increase glucose metabolism of the hypothalamus, caudate nucleus, cerebellum, temporal lobe, central gyrus, and brain stem (Mazoyer et al., 2001). Acupuncture at ST36 also induced an integrated response from multiple levels of the brain, including the anterior cingulate cortex (ACC), ventrolateral prefrontal cortex (VLPFC), supplementary motor area (SMA) primary/secondary somatosensory cortex (SI/SII), occipital cortices and midbrain (Hui et al., 2005; Liu et al., 2010). Therefore, acupuncture ST36 is connected with some brain areas. However, we need additional neurological techniques to study the effects of acupuncture on the brain.

Transcranial magnetic stimulation (TMS) is a noninvasive technique for neurological detection and treatment. TMS is used to measure the excitability and inhibition of the human motor cortex. For example, motor‐evoked potential (MEP) reflects the overall excitability of the cortex, spinal, and corticospinal (Ziemann & Rothwell, 2000). Short‐interval intracortical inhibition (SICI) and long‐interval intracortical inhibition (LICI) are the most common and well‐studied intracortical circuits in the primary motor cortex (M1); SICI and LICI are well‐established paired‐pulse TMS (pTMS; Chen, 2004). SICI refers to the phenomenon that a subthreshold conditioning stimulation (CS) that suppresses the MEP induced by subsequent suprathreshold test stimulation (TS) at interstimulus intervals (ISIs) of 1–5 ms. LICI is generated when a superthreshold CS is prior to the TS at ISIs of 50–200 ms (Müller‐Dahlhaus, Liu, Ziemann, & Florian, 2008).

Short latency intracortical inhibition and LICI are mediated by two types of gamma‐aminobutyrate (GABA) receptors: GABA_A_ and GABA_B_ (Wassermann et al., 1996). GABA is a major inhibitory neurotransmitter in the adult mammalian brain and is more widely distributed within the CNS. These neurotransmitters have been more extensively targeted for research and therapeutic application (McDonnell, Orekhov, & Ziemann, 2006; Watanabe et al., 2002). GABA exerts its inhibitory effect through the heterogeneity of its subunits; and the regulation of various intrinsic and extrinsic molecular factors. The interaction between the GABA receptor and the cytoskeleton appears to be important for the formation of neural networks. In addition, GABA is also involved in cell proliferation, differentiation, and growth. These roles are independent of neurotransmitter function (McDonnell et al., 2006; Watanabe et al., 2002).

There are four published studies that explore the impact of acupuncture on brain excitability by using TMS. Three studies showed that acupuncture is somatosensory stimulation that can induce significant MEP amplitude (Lo & Cui, 2003; Maioli, Falciati, Marangon, Perini, & Losio, 2006; Yew, Cui, & Fook‐Chong, 2005). One study showed that intracortical inhibition and intracortical facilitation were not modulated by acupuncture (Zunhammer, Eichhammer, Franz, Hajak, & Busch, 2012). Different acupuncture acupoints may generate different effects on the motor system. More acupoints should be investigated to ensure the effects on motor cortical excitability and inhibition. The effect of acupuncture at ST36 on motor cortical excitation and inhibition is still unclear.

In this study, we selected ST36 and its sham point and used single and paired TMS to measure MEP, SICI, and LICI during acupuncture. ST36 is used to treat dyskinesia and may be related to the recovery of the motor cortex (Watson, 2009). In addition, neuroimaging findings have shown that acupuncture at ST36 is associated with the supplementary motor area (Mazoyer et al., 2001). Therefore, we hypothesized that acupuncture at ST36 could increase motor cortical excitation and reduce motor cortical inhibition (SICI, LICI).

## 2 MATERIALS AND METHODS

### 2.1 Subjects

Twenty healthy, right‐handed subjects (mean age, 21.0 ± 4.4 years; 13 males and seven females) were recruited for participation in this experiment. All subjects provided informed consent prior to experimentation. All subjects were not contraindicated to TMS, had no neurological or cardiovascular disease, had no metal substances in the body, and had not undergone acupuncture in the month before commencing the study. This experimental procedure was approved by the local ethics committee of the Shanghai University of Sport.

### 2.2 Acupuncture methods

**Figure 1:**
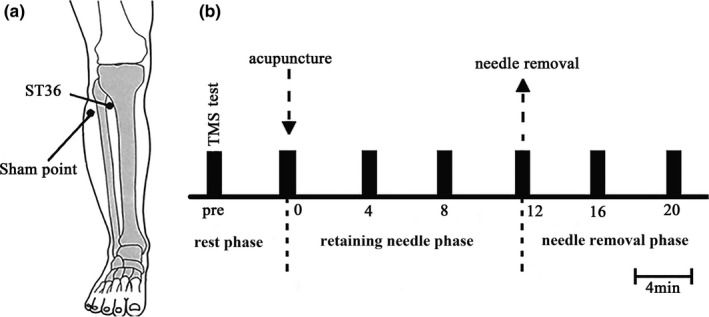
The location of Zusanli (ST36) and experimental procedure. The location of Zusanli (ST36) and experimental procedure. (a) Zusanli (ST36) located in the tibialis anterior muscle, four finger breadths of subject below the kneecap, and one finger breadth of subject lateral from the anterior crest of the tibia, and the sham point next to the ST36 about 3 cm as a control. (b) Experimental procedure, we evaluated MEP amplitudes at 7 time points, namely, before acupuncture (Pre), acupuncture (T0), 4 min after acupuncture (T4), 8 min after acupuncture (T8), needle removal (T12), 4 min after needle removal (T16), and 8 min after needle removal (T20). T0, T4, and T8 were the retaining needle phase, T12, T16, and T20 were the needle removal phase

ST36 is located at the tibialis anterior muscle four finger breadths of subject below the kneecap and one finger breadth of subject lateral from the anterior crest of the tibia (Figure 1a). The sham point located proximately 3 cm lateral to the ST36 and located between the bladder meridian and gallbladder meridian in TCM (Liu et al., 2010). ST36 and the sham point are all on the anterior tibia muscles, but not in the same meridians kin area. The entire experiment was carried out by the same clinician acupuncturist. Disposable sterile stainless steel needles (Wuxi, China) with a diameter of 0.22 mm and length of 40 mm were used for treatment. The needles were inserted vertically to a depth of 2–3 cm and subsequently rotated bidirectionally with an amplitude of approximately 180° at the rate of one cycle per second.

During acupuncture, the subjects were required to score their Deqi sensation by using a Gas table (10 VAS scale; Shi et al., 2014). The Gas table included scales on soreness, numbness, fullness, heaviness, and pain. Subscale assessment was as follows: 1–3, no or little discomfort; 4–6, light discomfort; 7–8, medium discomfort; 9–10, extreme discomfort; and >10, unbearable pain.

### 2.3 Electromyographic recording

The EMG signal was amplified (1,000 magnification) via DS7a amplifier (Canada) at a digitized sampling rate of 2 kHz, filtered via a CED (micro1401 laboratory interface, Cambridge Electronic Design), and then fed into a laboratory computer. Spike2 software (Version 8.0, Cambridge Electronic Design) was used for analysis. MEPs were recorded from the right first dorsal interosseous (FDI) muscles by surface electromyography using Ag–AgCl electrodes in a belly‐tendon montage of the FDI muscle. FDI muscle starts from the muscle belly of the first and second metacarpal bones and ends at the temporal side of the base of the proximal phalanx of the index finger. The nerve that governs FDI is the deep branch of the ulnar nerve.

### 2.4 Transcranial magnetic stimulation

In a quiet laboratory environment, the subjects were asked to wear rubber earplugs and goggles to reduce noise. The subjects were then instructed to remain in a comfortable sitting position, with their muscles in a completely relaxed state and without thinking activity. Single and paired‐pulse TMS (Magstim200) with a 70‐mm‐diameter coil was employed for testing. The coil was positioned flat on the head of the subjects at an angle of 45° from the midline and with the handle pointing backwards. First, we identified and marked any hot spot with a marker on the head. A hot spot was defined as a location with the largest and most consistent MEP.

Next, the resting motor threshold (RMT), active motor threshold (AMT), and 1 mV intensity were assessed for each subject. RMT was determined as the minimum stimulus intensity needed to elicit a MEP amplitude of ≥50 µV in at least five out of 10 consecutive trials (Wassermann et al., 1996). AMT was obtained during a slight isometric contraction (5%–10% of maximum voluntary contraction), and a MEP amplitude of ≥100 µV was elicited in at least five out of 10 consecutive trials (Romero, Anschel, Sparing, Gangitano, & Pascual‐Leone, 2002). One millivolt intensity was defined as the minimum stimulator output that generated MEP of more than 1 mV in at least five out of 10 consecutive trials. Ten consecutive magnetic stimulations were performed with an interval of 5–10 s. Finally, the average intensity was computed and used for further analysis.

### 2.5 Experiment 1

The experimental protocol is presented in Figure 1b. Acupuncture was performed in 20 subjects at ST36 and sham point in a random sequence. RMT intensity in FDI muscle was assessed for each subject before acupuncture, and then, we evaluated MEP amplitude from the right FDI muscle with stimulator output of 120% RMT (Maioli et al., 2006). Then, acupuncture was performed at ST36 on the right side, and the needle was removed after 12 min. During this process, we evaluated MEP amplitudes at 7 time points, namely, before acupuncture (Pre), acupuncture (T0), 4 min after acupuncture (T4), 8 min after acupuncture (T8), needle removal (T12), 4 min after needle removal (T16), and 8 min after needle removal (T20). T0, T4, and T8 were the retaining needle phase, T12, T16, and T20 were the needle removal phase. Six out of 20 subjects also underwent acupuncture at the left ST36 and sham point in a random sequence. The test methods and indicators were the same as at ST36 on the right side.

### 2.6 Experiment 2

Acupuncture was performed on 20 subjects at ST36 and sham point in a random sequence. Acupuncture at ST36 was performed on the right side, and the needle was removed after 12 min. RMT, AMT, and 1 mV intensity were assessed in each subject at three time points, before acupuncture (Pre), acupuncture (T0), and needle removal (T12). Then, two different intracortical inhibitory circuits (SICI and LICI) were observed at three time points. The CS of SICI was set at approximately 70% and 80% AMT, and TS was set to produce 1 mV MEP at an interval of 2 ms (Müller‐Dahlhaus et al., 2008). The CS of LICI was set at approximately 120% RMT, and TS was set to produce 1 mV MEP at an interval of 50 and 100 ms (Wassermann et al., 1996). Due to different experimental conditions that will change the index, to adjust TS to achieve 1 mV MEP amplitudes in different conditions. Motor cortical inhibition for each trial was expressed as the ratio between the mean conditioned and unconditioned MEP amplitude for each subject.

### 2.7 Data and statistical analyses

Spike2 software (CED) was used to collect all experimental data, which were exported to an Excel spreadsheet. Values were expressed as mean ± *SE*. SPSS 22.0 (IBM) was used for data processing. The normality of data distributions was examined with Kolmogorov–Smirnov tests. The VAS index was compared between stimulation groups using a paired *t* test. Two‐way repeated‐measures ANOVA was used to compare MEP before and after the intervention using time points (T0, T4, T8, T12, T16, T20) and acupuncture points (ST36, sham point) as independent factors. Two‐way repeated‐measures ANOVA was used to compare SICI and LICI before and after the intervention using time points (Pre, T0, T12) and acupuncture points (ST36, sham point) as independent factors. Significant effects and interactions were further investigated using Bonferroni‐corrected post hoc tests. Correlations between Deqi sensation and MEP were tested using Pearson product‐moment correlation coefficients. *p* < .05 was considered to represent statistical significance.

## 3 RESULTS

### 3.1 Deqi sensation

One subject did not complete the experiment due to scheduling difficulties. All subjects had no adverse reactions during the study. After acupuncture at ST36 or sham point, the subjects indicated the intensity of different Deqi sensations on a VAS index scale. The average results for each Deqi component (soreness, aching, deep Pressure, heaviness, fullness, tingling, numbness, sharp pain, dull pain, warmth, cold, throbbing, other) are presented in Table 1. Statistical analysis showed that average Deqi sensation had a greater intensity for ST36 (5.01 ± 1.10) than for the sham point (2.33 ± 0.90; *p *< .01, Table 1).

**Table 1:** Deqi sensation

Comparison of intensity ratings and VAS Index when acupuncture (Mean ± *SE*)		
Deqi sensation	ST36	Sham point
Aching	5.23 ± 1.42**	2.70 ± 1.50
Soreness	6.00 ± 1.77**	2.88 ± 0.90
Numbness	5.35 ± 2.53**	1.82 ± 1.30
Fullness	6.82 ± 1.33**	2.82 ± 1.71
Heaviness	4.76 ± 2.60**	2.00 ± 1.18
Dull pain	4.59 ± 2.66**	2.29 ± 1.27
Sharp pain	2.59 ± 1.20*	1.18 ± 0.78
Tinging	5.00 ± 1.54**	1.94 ± 1.24
Pressure	5.35 ± 1.18**	2.00 ± 1.10
Warmth	3.11 ± 1.58**	1.17 ± 0.97
Coolness	1.70 ± 1.07*	1.47 ± 0.82
Throbbing	2.76 ± 1.50**	1.00 ± 0.66
Other	0.53 ± 0.51	0.47 ± 0.61
VAS index	5.01 ± 1.10**	2.33 ± 0.90

Note

Mean ± *SE*.

* *p* < .05.

** *p* < .01, ST36 compared with sham point.

### 3.2 Experimental 1

**Figure 2:**
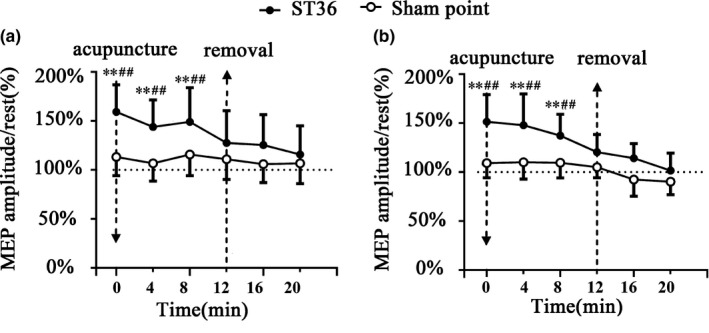
MEP amplitude during acupuncture ST36 and sham point. MEP amplitude during acupuncture right ST36 and sham point in seven time points. (a) Acupuncture right ST36 and sham point. (b) Acupuncture left ST36 and sham point. Compared the rest MEP amplitude (Pre) with the other time points: *p < .05, **p < .01, there were significant differences in T0, T4, and T8. Compared the MEP amplitudes of ST36 with sham point at the same time point: #*p* < .05, ##*p* < .01, there were significant differences in T0, T4, and T8

**Figure 3:**
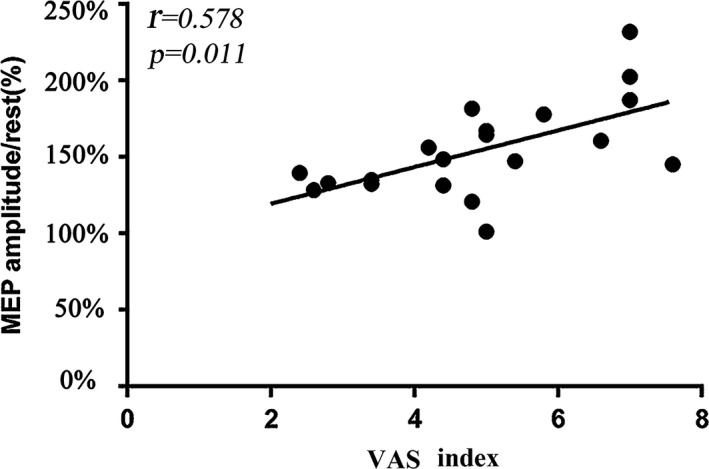
Correlation between Deqi sensation and cortical excitation. The abscissa indicates the VAS index (Deqi sensation). VAS index and cortical excitation, the coordinate indicates the value of MEP amplitudes. Solid lines represent the significant correction between VAS index and MEP amplitudes when acupuncture at ST36 and sham point

MEP amplitude at 7 time points during acupuncture at ST36 or the sham point is presented in Figure 2. MEP amplitude at the Pre was similar between acupuncture ST36 (0.25 ± 0.05) and the sham point (0.24 ± 0.04). As in Figure 2, MEP amplitude in Pre was used as the baseline value for further comparison. When acupuncture at ST36, MEP at T0, T4, and T8 were significantly increased compared with baseline (*p *< .05, Figure 2a). When acupuncture at the sham point, MEP was not significant (*p* < .05, Figure 2a). MEP at T0, T4, and T8 was significantly increased between acupuncture at ST36 and the sham point (*p* < .05, Figure 2a). We investigated the relationship between the VAS index and cortical excitation. The correlation between Deqi sensation score and MEP amplitudes had significant differences at T0 (*r* = .578, *p* = .011, Figure 3).

With acupuncture at ST36 on the left side, the MEP at T0, T4, and T8 showed significant differences compared with that at Pre (*p* < .05, Figure 2b). Such significance was also detected at T0, T4 and T8 compared with that at the sham point (*p* < .05, Figure 2b). The statistical results were similar with those undergoing acupuncture ST36 on the right side.

### 3.3 Experimental 2

When acupuncture at ST36, RMT, AMT, and 1 mV in T0 were lower than these at Pre (Table 2). Importantly, when acupuncture at ST36, RMT, AMT, and 1 mV at T0 were lower than those at the sham point (*p* < .05, Table 2). Test stimulus MEP and conditioned stimulus MEP of SICI and LICI at 3 time points are presented in Table 2. We valued SICI and LICI by using conditioned stimulus MEP over test stimulus MEP (% of TS alone). We found that SICI_70% AMT_ (% of TS alone), SICI_80% AMT_ (% of TS alone), LICI_50 ms_ (% of TS alone), and LICI_100 ms_ (% of TS alone) at Pre were similar between acupuncture ST36 and sham point.

**Table 2:** Transcranial magnetic stimulation (TMS) measurements

Measurements	ST36			Sham point		
	Pre	T0	T12	Pre	T0	T12
RMT (% MSO)	40.25 ± 4.37	35.36 ± 5.46*,**	37.97 ± 7.5	40.3 ± 6.18	38.64 ± 7.9	39.35 ± 7.26
AMT (% MSO)	25.32 ± 5.07	21.56 ± 3.0*,**	23.87 ± 5.08	26.52 ± 5.37	24.46 ± 6.19	24.95 ± 4.84
1 mV (% MSO)	72.35 ± 6.74	61.55 ± 4.61*,**	75.82 ± 8.33	73.12 ± 7.38	70.23 ± 8.69	69.42 ± 7.52
TS alone/mv	1.08 ± 0.12	1.10 ± 0.06	1.05 ± 0.15	1.07 ± 0.18	1.04 ± 0.07	1.05 ± 0.16
SICI_70% AMT_/mv	0.27 ± 0.04	0.40 ± 0.05	0.30 ± 0.03	0.27 ± 0.03	0.33 ± 0.04	0.28 ± 0.03
SICI_80% AMT_/mv	0.18 ± 0.03	0.28 ± 0.04	0.21 ± 0.08	0.18 ± 0.04	0.21 ± 0.04	0.21 ± 0.03
SICI_70% AMT_ (% TS alone)	25.04 ± 4.07	36.36 ± 5.97	28.42 ± 4.20	25.27 ± 4.10	31.82 ± 4.97	26.56 ± 3.04
SICI_80% AMT_ (% TS alone)	16.89 ± 3.09	25.08 ± 4.84	20.34 ± 3.06	17.06 ± 3.97	20.21 ± 4.90	19.91 ± 3.75
LICI_50 ms_/mv	0.37 ± 0.05	0.60 ± 0.04	0.36 ± 0.04	0.36 ± 0.05	0.41 ± 0.03	0.36 ± 0.05
LICI_100 ms_/mv	0.28 ± 0.05	0.42 ± 0.05	0.29 ± 0.03	0.27 ± 0.05	0.30 ± 0.06	0.29 ± 0.03
LICI_50 ms _(% TS alone)	31.52 ± 6.50	50.14 ± 5.46	34.47 ± 6.90	31.81 ± 3.20	38.14 ± 6.74	32.36 ± 4.86
LICI_100 ms _(% TS alone)	24.46 ± 3.91	34.75 ± 3.69	25.58 ± 4.86	24.69 ± 4.21	29.20 ± 5.13	26.02 ± 4.35

Note

Mean ± *SE*.

Abbreviations: AMT, action motor threshold; LICI, long latency intracortical inhibition; MSO, maximum stimulator output; RMT, resting motor threshold; SICI, short latency intracortical inhibition; TS (alone), test stimulus MEP.

* *p* < .05, compared with different time.

** *p* < .05, ST36 compared with sham point.

**Figure 4:**
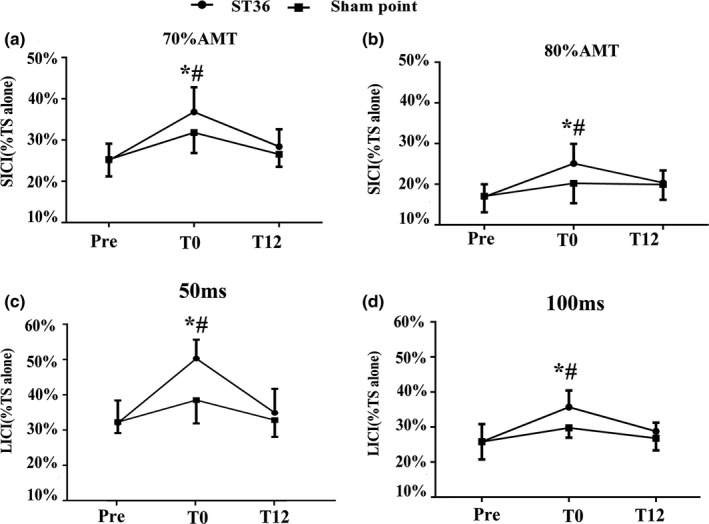
Motor cortical inhibition when acupuncture left ST36 and sham point in three time points. Motor cortical inhibition during acupuncture left ST36 and sham point in three times (Pre, T0, T12). SICI and LICI were measured using conditioned stimulus MEP over test stimulus MEP. (a) SICI (%TS alone), the CS intensity is respectively 70% AMT. (b) SICI (%TS alone), the CS intensity is respectively 80% AMT. (c) LICI (%TS alone), the ISI is 50 ms. (d) LICI (%TS alone), the ISI is 100 ms. Compared the same position in different time point: *p < .05, there were significant (*p* < .05) differences in T0 compare with Pre. #*p* < .05, there were significant (*p* < .05) differences in acupuncture at ST36 compare with the sham point in T0

As in Figure 4, SICI (% of TS alone) and LICI (% of TS alone) at three time points are represented by a line graph. When acupuncture at ST36, SICI_70% AMT_ (% of TS alone), and SICI_80% AMT_ (% of TS alone) showed a significant difference between T0 and Pre (*p* < .05, Figure 4a,b), SICI_70% AMT_ (% of TS alone) and SICI_80% AMT_ (% of TS alone) at T0 also showed a significant difference compared with those at the sham point (*p* < .05, Figure 4a,b). When acupuncture at ST36, LICI_50 ms_ (% of TS alone), and LICI_100 ms_ (% of TS alone) also displayed a significant difference between T0 and Pre (Figure 4c,d). LICI_50 ms_ (% of TS alone) and LICI_100 ms_ (% of TS alone) in T0 also showed a significant difference compared with those at the sham point (*p* < .05, Figure 4c,d).

## 4 DISCUSSION

To further investigate the neural effects of acupuncture, we studied the effects of acupuncture at ST36 on motor cortical excitation and inhibition by TMS. Our study found that acupuncture at ST36 increased motor cortical excitation compared with the sham point. Deqi sensation was correlated with MEP. Deqi sensation may be important for the effect on motor cortical excitability when acupuncture at ST36. Acupuncture at ST36 also reduced motor cortical inhibition (SICI and LICI).

### 4.1 Deqi sensation during acupuncture at ST36 or sham point

To study the efficacy of acupuncture in TCM, a sham point is often used for comparative research. An ideal sham point should be located a certain distance away from the known acupuncture point, but not in the same meridian (Maioli et al., 2006). In this experiment, we selected the sham point that was approximately 3 cm from the ST36. The sham point was selected in accordance with the principle of selecting sham point from the middle line between the two meridians commonly used in TCM studies (Jung et al., 2016).

Acupuncture at ST36 was observed to elicit a stronger Deqi sensation than sham point. The complex pattern of sensations in the Deqi response suggests the involvement of a wide spectrum of myelinated and unmyelinated nerve fibers, particularly slower conducting fibers in the tendinomuscular layers (Hui et al., 2007). Local anesthesia at ST36 can block most Deqi sensations and inhibit brain responses to Deqi (Jin et al., 2014). Real acupuncture and sham points display a significant overlap in the active brain regions, but the extent and location of the active regions differ (Yoo et al., 2007). Acupuncture points may modulate somatosensory and saliency processing regions more readily than those of stimulation at sham points (Nierhaus et al., 2015).

### 4.2 Effects of motor cortical excitation during acupuncture at ST36 or sham point

Motor‐evoked potentials amplitudes are predominantly influenced by changes in synaptic excitability, as evidenced by alterations in the presence of pharmacological modifiers for synaptic transmission (Boroojerdi, Battaglia, Muellbacher, & Cohen, 2001). The current findings showed that acupuncture at ST36 led to changes in cortical excitability. Similarly, acupuncture at Hegu and Shou‐sanli can modulate cortical excitability (Lo & Cui, 2003; Yew et al., 2005). Acupuncture at ST38 (Tiaokou) also exerts an effect on motor cortical excitability (Maioli et al., 2006). These acupoints are basically on the limbs and can be used clinically for analgesia. Acupuncture of these acupoints also produce very mild, local, and long‐lasting somatosensory stimulation and could be enough to induce and regulate the excitability of the motor cortex. From previous neuroimaging studies, acupuncture can be seen to specifically activate corresponding brain regions, and these brain regions are closely related to acupoints. The motor cortex may be the area they are coactivating (Jiang et al., 2013; Kong et al., 2009; Li et al., 2008; Liu et al., 2013; Mazoyer et al., 2001; Sun et al., 2012). For example, acupuncture at ST36 increases the glucose metabolism of the hypothalamus, caudate nucleus, cerebellum, temporal lobe, central gyrus, and brain stem (Mazoyer et al., 2001). This effect increased blood flow and oxygen content may have a relationship with cortical excitability.

Traditional Chinese medicine contends that the therapeutic effect of acupuncture is transmitted through 12 meridians. When we performed acupuncture at the left‐sided ST36, the effects on cortical excitability were similar to the right ST36, in other words, acupuncture also influenced the excitability of the ipsilateral hemisphere. Therefore, acupuncture of the left side of the body can also regulate motor cortex excitability of the left brain. A reasonable explanation for this excitability correlation is the transcallosal pathway model. This model contends that the inhibitory effect from right M1 (contralateral to acupuncture side) to the left M1 (ipsilateral to acupuncture side) declined significantly after acupuncture intervention. Since the ipsilateral M1 received less inhibition, excitability increased (Yang et al., 2017). This might be the explanation for how acupuncture modulates the excitability of the ipsilateral M1.

### 4.3 Correlation between Deqi sensation and cortical excitation

In this study, Deqi sensation was observed to be correlated with cortical excitation. Specifically, stronger Deqi sensation had stronger MEP amplitude. We speculated that Deqi sensation is important for increasing motor cortical excitation. Furthermore, Deqi sensation may change the excitation of motor neuron pools. Yang et al. (2017) found that corticomotoneuronal excitability and interhemispheric competition could be modulated by acupuncture therapy in healthy subjects. Acupuncture stimulation may generate physiological effects on the autonomic nervous system via the activation of a somatosensory pathway (Kim, Park, & Namgung, 2012). Therefore, Deqi sensation may be the key to the effect on motor cortical excitability when acupuncture at ST36.

### 4.4 Changes in motor cortical inhibition during acupuncture at ST36

Cortical inhibitory circuit plays a major role in the modulation of motor outputs from M1. The motor cortex can be inhibited by both intracortical mechanisms and by peripheral sensory inputs. SICI is mediated by GABA_A_, and LICI is mediated by GABA_B_ (Wassermann et al., 1996). The results demonstrated that acupuncture at ST36 decreased SICI and LICI compared with Pre. As acupuncture provides somatosensory conditioning stimulus, mixed, or cutaneous input from the hand can suppress the excitability of the motor cortex at short latency (Tokimura et al., 2000). Somatosensory input may decrease the concentration of GABA_A_ and GABA_B_ receptors within the motor cortex, thereby decreasing SICI and LICI. Zunhammer et al. (2012) found that needling GB34 exerted no significant effects on SICI. This result may be attributed to acupuncture point specificity. The results of this study compared to past research support the hypothesis that acupuncture at different acupoints is associated with different effects on motor systems evoked by acupuncture stimulation. Therefore, other acupuncture points should be investigated to empirically characterize their effects on motor cortical inhibition.

### 4.5 Limitations

This study has some limitations that should be noted. Only one acupoint was tested. Although a single acupuncture point can be used to explain a therapeutic effect, and analysis of more acupuncture points would provide more convincing evidence. Additionally, we only analyzed the effect of acupuncture on motor cortical excitation and inhibition in healthy people, which may be different under different conditions. It would be enlightening to execute a study subjects who are in a fatigued or diseased condition in the future. Further investigations should be performed with a particular emphasis on the measurements for resting motor threshold, cortical silent periods, and intracortical facilitation.

## 5 CONCLUSION

In summary, the present study suggested that acupuncture at ST36 modulates cortical excitation and had an effect on the remaining needle phase. Resulting Deqi sensation was correlated with cortical excitation when acupuncture was performed at ST36. Acupuncture at ST36 also decreased cortical inhibition compared to sham point. These results provided additional evidence for the efficacy of acupuncture.

## CONFLICT OF INTEREST

The authors declare no conflicts.

## Data Availability

The data are not publicly shared due to privacy.
